# Chicago Veterinary College

**Published:** 1887-07

**Authors:** 


### CHICAGO VETERINARY COLLEGE.

The fourth annual commencement exercises of the Chicago Veterinary College were held March thirty-first in the new college building, Nos. 2357 and 2359 State street. The President's address was a review of the foundation, growth and requirements of the institution. The whole number of matriculants was fifty-three. The graduating class consisted of the following twenty-six members : E. M. Heath, of Massachusetts ; J. D. Robinson, of Ontario; E. L. Kennedy, of Michigan ; D. R. Moysey, of Ohio ; A. W. Dreier, of Wisconsin ; J. M. Phillips, of Illinois ; G. R. Young, of Nebraska ; E. R. Voorhees, of New Jersey ; T. G. Sabin, Massachusetts; S. S. Snyder, Ohio ; Chas. W. Johnson, Illinois ; J. H. Wattles, Wisconsin ; J. T. Clark, Iowa ; F. N. Rowan, Illinois ; Gilbert Hess, M. D., Ohio ; F. T. Eisenman, Kentucky ; W. S. Brayton, Illinois ; D. R. Price, Wisconsin ; H. H. Tarr, New York ; A. Cooley, Ohio ; R. C. Moore, Kansas ; L. S. Pocock, Colorado ; A. G. Bernard, Illinois ; F. G. Harbour, Illinois ; L. F. Brown, Dakota ; G. H. Smith, Illinois. After the conferring of the degree of Doctor of Veterinary Science on the graduates by the President, an able valedictory was read by Dr. Chas. W. Johnson.

He dwelt on the trials and difficulties of the student, comparing the pains of the past, with the pleasures of the present moment of success in holding the diploma which entitled them to the honors and dignities of Veterinary Surgeon.

He pictured to the minds of his fellow graduates, the vast field which their adopted profession opened before them, of labor and struggles, of success and fame. He pointed out the deficiencies of its literature, and their obligations to correct them, the low standing of most of its members, and the means of raising it. He urged them to systematize their work and never to lag in the road of progress, but push on with a surety of a brilliant harvest. He appealed to them to remember always their Alma Mater with pride, and their professors with gratitude.

Dr. E. M. Reading, Professor of Physiology, delivered the doctorate address, in which he demonstrated the close relationship which exists between Human and Veterinary Medicine. "Human Physiology," he says, "is based largely upon the study of animals, while Animal Chemistry applies equally to them as to man." He showed how the same might be said, with a little modification, of the other branches, and saw no good reason why a Veterinarian should not be a good Human Physician, and *vice versa*.

The Doctor dwelt on the fact that Veterinary Science is less highly esteemed than its sister profession ; that the latter has been thousands of years in its growth, while the former has occupied only a century in reaching its present standing, and said: "Age always inspires veneration, apart from a consideration of real merit. The science of Astronomy, which certainly dates back to the building of the pyramids, we regard with wonder, while chemistry can show a history of only two hundred or three hundred years, yet the latter science is far more serviceable to mankind. In like manner, Veterinary Medicine lacks age, and consequently inspires less reverence than its sister profession, but without reason, as we should esteem all human institutions for what they are, rather than what they have been."

Attention was called to the number of ignorant "horse doctors," but this fact was shown to be no more proof of the low character in the Veterinary profession, than that the number of quacks in the human profession, was also a mark of its discredit. Again, the Veterinarian has a greater field for investigation, as his efforts tend to make the science of medicine more exact through experimental means. He has the advantage also, in that he need not hesitate to sacrifice life, in order to demonstrate a scientific truth, or evolve a new law.

*Apropos* of the necessity of high mental qualities in the Veterinarian he said :—"Thus do I say that in the Veterinary branch of Medicine, where the field for investigation is practically unlimited, we need men of learning and high mental endowments, men, whose discrimination is exact, whose perception is clear, whose logic is sound and whose conclusions are well-founded.

"If Science is ennobling, if it develops culture and refinement, then ought the learned Veterinarian be honored among men.

"This institution, just turning four years of age, is rapidly gaining the good reputation that it so well deserves. This year's graduating class may well be proud of their Alma Mater, and the Faculty have good reason to expect more than average success from this class, for a more intelligent, studious lot of gentlemen never went out of any college. As an evidence of this fact, and of the ability of the Faculty to impart their knowledge to others, we may point to the fact that fourteen out of the twenty-six graduated, passed with honors."

The advantages possessed by the Chicago Veterinary College, are many. The new building, 50x146 feet is located on the principal street in the city, has a reception room, private offices for the officers of the corporation, pharmacy, carriage floor, bath-room for patients, store-room for slings, hobbles, etc., a large hospital, well lighted and ventilated, with commodious box stalls, and a large operating-room on the ground floor. The lecture hall, dissecting room, museum, dog kennels and feed-room, are on the second floor.

The hospital is sufficiently appreciated by the public to keep it full all the time. The senior students are appointed to dresserships and pharmacy work during the session, which familiarizes them with the practical application of the theory taught in the lectures. They are allowed also to assist at operations. The existence of contagious pleuro-pneumonia in Chicago last fall gave an opportunity for the students to get clinical lectures on the disease, and to make examinations ante mortem and post mortem in all stages and forms of the disease.

